# Severe cytokine release syndrome induced by immune checkpoint inhibitors in cancer patients – A case report and review of the literature

**DOI:** 10.1016/j.heliyon.2024.e24380

**Published:** 2024-01-10

**Authors:** Yujing Zhang, Xiaoyue Wen, Yaqi OuYang, Yingying Hu, Xiangzhi Fang, Jiancheng Zhang, Yin Yuan

**Affiliations:** a Department of Critical Care Medicine, Union Hospital, Tongji Medical College, Huazhong University of Science and Technology, Wuhan, 430022, PR China; b Institute of Anesthesia and Critical Care Medicine, Union Hospital, Tongji Medical College, Huazhong University of Science and Technology, Wuhan, 430022, PR China

**Keywords:** Immunotherapy, Immune checkpoint inhibitor, Immune-related adverse event, Cytokine release syndrome, Case report

## Abstract

Cytokine release syndrome (CRS) can be induced by immune checkpoint inhibitors (ICIs). Although the incidence of CRS is low, it is often underreported. Here, we report two severe CRS cases and summarize and review 51 patients with ICI-induced CRS to explore the possible contributing factors to the disease prognosis and provide assistance for therapy. Our analysis found that the population with ICI-induced CRS consists mainly of male patients with an average age of 61.74 years. The primary malignant tumor type was lung cancer, and the clinical stage of most patients was stage IV. Notably, patients who experience a longer time to CRS onset, higher IL-6 levels, and lower platelet counts may be more likely to develop severe CRS. Cardiovascular, respiratory, neurological, and coagulation toxicities are more common in higher-grade CRS and may serve as markers for patient experiencing ICU admission, oxygen supplementation, hypotension, high-dose vasopressors usage, and intubation.

## 1 Introduction

Cancer is one of the most lethal maladies worldwide. In recent times, there have been remarkable advancements in cancer therapy through immunotherapy. The immune checkpoint inhibitors (ICIs) are a unique category of immunotherapy medications that work by obstructing key regulatory signals that impede immune responses. The American Food and Drug Administration has sanctioned two main classes of ICIs that are employed clinically: programmed cell death receptor-1 (PD-1) pathway inhibitors, including PD-1 inhibitors and PD-1 ligand 1 (PD-L1) inhibitors, and anti-cytotoxic T lymphocyte-associated antigen (CTLA)-4 inhibitors (CTLA-4 inhibitor) [1]. Even though study has demonstrated that ICIs have dramatically improved the prognosis of many cancer patients in many clinical situations, such as melanoma, renal cell carcinoma, non-small cell lung cancer (NSCLC), bladder cancers, Hodgkin lymphoma, and others [2], they are closely associated with an extensive spectrum of immune-related adverse events (irAEs). IrAEs are differ from those arising from chemotherapy and targeted drug and have been reported to occur in nearly every organ system, including dermatitis, arthritis, colitis, hepatitis, myocarditis, pneumonitis, encephalitis, and others [3,4]. Cytokine release syndrome (CRS) can occur as an irAE, which was initially described in the early 1990s when the anti-T-cell antibody muromonab-CD3 was utilized as an immunotherapy for solid organ transplantation [5]. The incidence of ICI-induced CRS is estimated to be about 0.07% [6]. Although CRS is considered rare, it can be severe and life-threatening. Early recognition of CRS and rapid initiation of appropriate treatment measures are crucial in preventing life-threatening sequelae, or even mortality. Several management guidelines have been established for ICI-related toxicities [3]. However, rare, complex, and life-threatening CRS remain a challenge for clinicians. Here, we present two cases of acute CRS that developed after the first intravenous dose of anti-PD-1inhibitor. Furthermore, we summarize and analyze existing reports on CRS, with the aim of exploring the characteristics and possible contributing factors of ICI-induced CRS and aiding clinicians in appropriately managing them.

## 2 Case presentation

### 2.1 Case one

A 74-year-old man was admitted to our hospital with persistent chest pain spanning a week. He had a medical history that comprised of a nineteen-year-long battle with liver cancer, forty years of tuberculosis, over two decades of well-controlled hypertension, and percutaneous coronary stent implantation three years ago. Upon admission, laboratory tests showed no signs of organ dysfunction. Further, a positron emission tomography/computed tomography (PET/CT) scan revealed the presence of a pulmonary tumor in the lower lobe of his left lung. The subsequent transbronchial biopsy confirmed poorly differentiated non-small cell lung cancer (NSLC) and did not indicate metastatic hepatocellular carcinoma. Clinically, the patient was in an advanced stage IIIA. The medical team determined that neoadjuvant chemoimmunotherapy would be the first course of action, to evaluate the potential of downstaging the patient for surgery. Subsequently, the patient was received a combination of 200 mg sintilimab and 400 mg nab-paclitaxel, without any untoward discomfort, with normal blood routine, hepatic, renal and cardiac functions at that time.

On the second day subsequent to the single dose of sintilimab and nab-paclitaxel administration, the patient exhibited dyspnea and gradually lapsed into unconsciousness with negative pathological signs. An emergency cranial and thoracic CT scan was promptly conducted and divulged no intracerebral hemorrhage and cerebral infarction (Fig. 1a–c), and the lungs, particularly the left lung, evinced pronounced atelectasis and multiple grid-like changes (Fig. 2). Thus, he was transferred to the intensive care unit (ICU) where arterial blood gas analysis evinced severe acidosis, significant carbon dioxide retention, and an oxygenation index of 133. He underwent emergent tracheal intubation with ventilator-assisted breathing. Bronchoscopy showed no airway obstruction. Meanwhile, white cell count, plasma procalcitonin level, β-1,3-glucan test, and galactomannan test were within normal range. Sputum microbial testing, including staining and culture for bacteria, fungi, and mycobacteria, also yielded negative results. Based on his clinical presentations and laboratory tests, immune-related pneumonia induced by sintilimab was suspected. The patient was received intravenous methylprednisolone (mPSL) 500 mg once daily for three days to alleviate inflammation, prone position ventilation to reduce atelectasis, acetylcysteine inhalation solution 300 mg every 8 h to minimize phlegm, budesonide inhalation suspension 1 mg every 12 hours to relax bronchial smooth muscle, cefotaxime sodium 2 g every 8 h to prevent infection, as well as nutritional support to improve clinical symptoms. On the second day of ICU admission, the patient regained consciousness and a chest CT scan was reviewed, revealing slight reduction in atelectasis and grid-like changes (Fig. 3). The pulse-dose mPSL was gradually tapered to 40 mg every 12 hours per day on the third day.

**Fig. 1:**
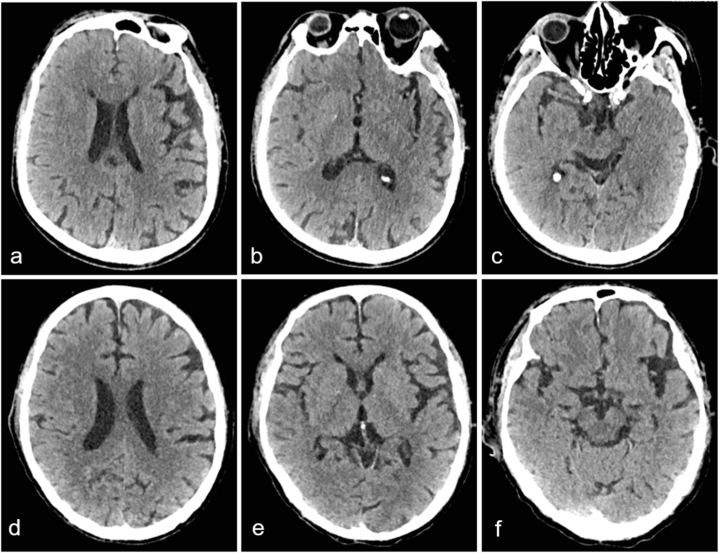
Brain CT scan on day 2 after sintilimab administration and on day 7 after ICU admission. (a–c) The brain CT on day 2 after sintilimab administration. (d–f) The brain CT on day 7 after ICU admission. CT, computed tomography; ICU, intensive care unit.

**Fig. 2:**
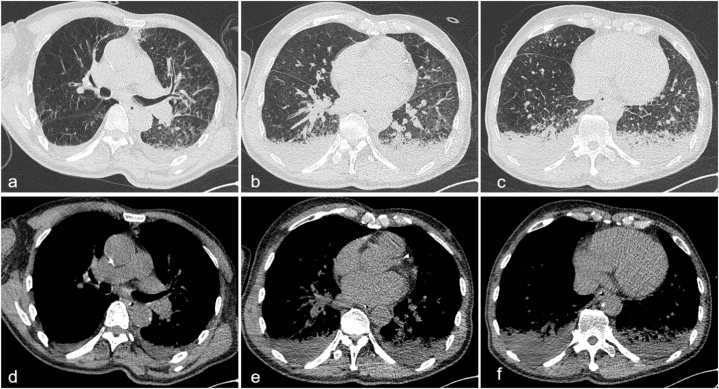
Emergency chest CT scan on the second day after sintilimab administration. (a–c) The lung window images. (d–f) The mediastinal window images. CT, computed tomography.

**Fig. 3:**
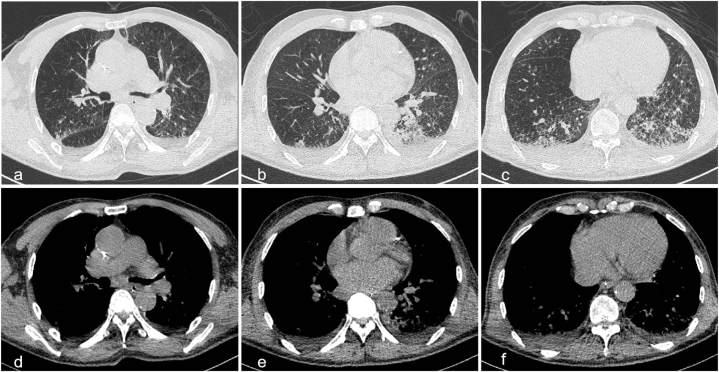
Repeat chest CT scan on day 2 after ICU admission. (a–c) The lung window images. (d–f) The mediastinal window images. CT, computed tomography; ICU, intensive care unit.

**Fig. 4:**
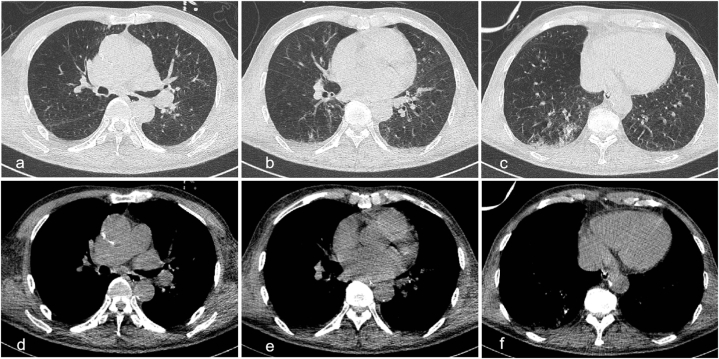
The chest CT scan on day 7 after ICU admission. (a–c) The lung window images. (d–f) The mediastinal window images. CT, computed tomography; ICU, intensive care unit.

The patient experienced myelosuppression following chemotherapy on the sixth day of ICU admission, with a rapid reduction in leukocytes and platelets to 1.90 × 10^9^/L and 50 × 10^9^/L, respectively. In addition, the patient developed atrial fibrillation and decreased urine output. Troponin I levels were slightly elevated at 58.5 ng/L, BNP levels were 124.3 pg/mL, and the serum creatinine levels were significantly elevated but within normal range. The patient's condition continued to deteriorate, with anuria and consciousness disturbance on the seventh day. Point-of-care ultrasound revealed a left ventricular ejection fraction of less than 40%, left atrial enlargement, and mild to moderate aortic and tricuspid regurgitation. Brain and chest CT scans showed no abnormality in brain (Fig. 1d–f) and no significant disease progression in the lungs (Fig. 4). Further serological investigations revealed increased levels of C-reactive protein (CRP) (max. 190 mg/L; N < 5 mg/L), interleukin (IL)-8 (226.30 pg/mL; N < 21.4 pg/mL), IL-17 (40.56 pg/mL; N < 20.6 pg/mL), IL-10 (8.57 pg/mL; N < 5.9 pg/mL), IL-5 (4.18 pg/mL; N < 3.4 pg/mL) and tumor necrosis factor (TNF)-α (10.35 pg/mL; N < 5.5 pg/mL). There was strong suspicion of CRS induced by sintilimab. The dose of mPSL was increased to 80 mg every 12 hours per day, and anti-infective drugs were empirically replaced by meropenem in combination with caspofungin. The patient was routinely supported with continuous blood purification to reduce the capacity load, inotropic drugs to enhance myocardial contraction, amiodarone and b-blocker to eliminate arrhythmia, and human granulocyte colony-stimulating factor (G-CSF) and thrombopoietin to promote leukocytes and platelet production, respectively. Unfortunately, due to financial burden, the patient was discharged on the eighth day and died shortly after returning home during a telephone follow-up.

### 2.2 Case two

The second patient was a 62-year-old man who diagnosed with oral cancer located at the floor of his mouth. His medical history included secondary pulmonary tuberculosis and hepatitis B virus (HBV) infection for 18 years and currently in stable phase, chronic bronchitis and pneumoconiosis for over 20 years with bronchiectasis, emphysema and bullae, as well as controlled hypertension for 10 years and a 40-year history of smoking. Upon admission, laboratory tests did not indicate any signs of organ dysfunction, with normal HBV-DNA levels and negative antibodies for other hepatitis viruses. The tuberculin test and sputum smear for mycobacteria detection were negative. The patient underwent stomatoplasty, and the biopsy revealed oral squamous cell carcinoma, evaluated as clinical stage IVA. Consequently, chemotherapy in combination with immunotherapy was administered 7 days later, consisting of 200 mg penpulimab, 400 mg nab-paclitaxel, and 40 mg cisplatin.

On day 3 after chemotherapy and immunotherapy, the patient developed chemotherapy-induced myelosuppression with a platelet count of 44 × 10^9^/L and gastrointestinal hemorrhage accompanied by melena. The instant blood tests revealed a significant drop in hemoglobin levels from 15 g/dL to 11 g/dL, while his liver and kidney function tests showed remarkably impairments, with an elevated serum level of alanine aminotransferase (ALT) at 750 U/L, aspartate aminotransferase (AST) at 900 U/L, total bilirubin at 86.5 μmol/L, and creatinine at 464.5 μmol/L. Additionally, his HBsAg tested positive, and serum HBV-DNA levels were 299 IU/mL. However, the patient refused a digestive endoscopy and was subsequently transferred to the ICU, where he received treatment consisting of octreotide acetate combined with terlipressin to reduce hemorrhage, fresh frozen plasma and platelets to improve clotting, entecavir to inhibit HBV replication, magnesium isoglycyrrhizinate and reduced glutathione to protect liver function, and meropenem to prevent infection. Fortunately, symptoms of gastrointestinal hemorrhage were quickly relieved.

On the second day of ICU admission, the patient’s condition precipitously worsened, resulting in circulatory collapse. Consequently, he required intubation and mechanical ventilation to assist with breathing, and high-dose norepinephrine (>1.0 μg/kg/min) was administered to maintain blood pressure. Point-of-care ultrasound revealed diffuse cardiac hypokinesia with left ventricular ejection fraction less than 30%. Troponin I and BNP levels were slightly elevated, measuring 50.5 ng/L and 141.2 pg/mL, respectively. Importantly, IL-6 serum levels were elevated >5000 pg/mL and IL-10 serum levels were 768.83 pg/mL. Sputum and blood microbial testing were negative, while white cell count was normal and plasma procalcitonin levels were 6.47 ng/mL. Based on the patient’s clinical presentation and laboratory tests, CRS induced by penpulimab was diagnosed. Intravenous mPSL 500 mg once a day was administrated to mitigate inflammation. However, the patient's clinical symptoms continued to deteriorate, with the electrocardiogram showing sustained atrial arrhythmia, complete right bundle branch block, and extensive ST-depression. Ultimately, the patient succumbed to refractory cardiogenic shock, experiencing recurrent hypotension, and died on the same day.

## 3 Literature review

### 3.1 Data collection

**Fig. 5:**
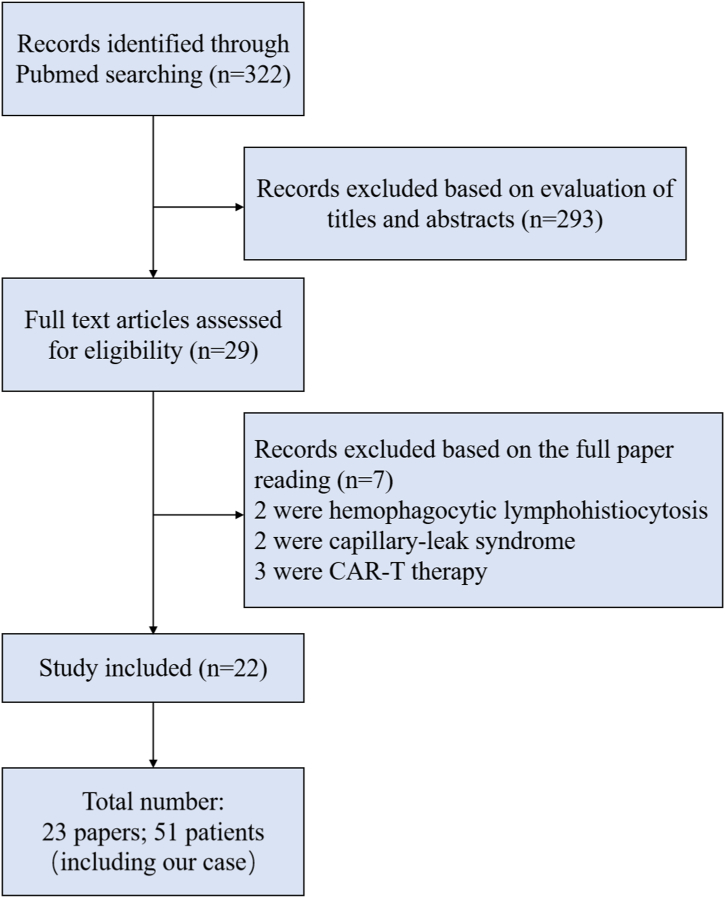
Screening process.

Through a rigorous exploration of the PubMed database using various search terms such as immune checkpoint inhibitors, immunotherapy, immune-related adverse events, cytokine storm, and cytokine release syndrome, we were able to identify a total of 322 articles. After carefully screening and eliminating duplicate cases and irrelevant reports, 29 papers were identified. Subsequently, an additional seven papers were excluded upon closer inspection, as they pertained to hemophagocytic lymphohistiocytosis, capillary-leak syndrome, or anti-CD19 chimeric antigen receptor (CAR) T-cell therapy. In total, we accurately summarized 22 papers [[7], [8], [9], [10], [11], [12], [13], [14], [15], [16], [17], [18], [19], [20], [21], [22], [23], [24], [25], [26], [27], [28], [29], [30]]. A total of 49 patients were enrolled. Fig. 5 showed the screening process. Supplementary Data presents basic information of 23 literatures and 51 patients who experienced ICI-induced CRS (including our present cases).

### 3.2 Statistical analysis

The data collected were analyzed using SPSS version 20.0 software (SPSS, Tokyo, Japan). Data were expressed according to their scaling as arithmetic mean ± standard error of mean (SEM) or frequencies [%]. Proportions for categorical variables were compared using the χ^2^ test, Yates’ continuity corrected χ^2^ test, or Fisher’s exact test. Continuous variables were compared using independent group t tests or the Mann-Whitney test. *P* < 0.05 was considered statistically significant.

### 3.3 Results

From the search results of all patient baseline characteristics (Table 1), we ascertained that the male accounted for a significant proportion (68.63%) of the population with ICI-induced CRS, with an average age of 61.74 years. The types of primary malignancies in our case series were lung cancer (43.14%), melanoma (11.76%), renal cell carcinoma (9.80%), hepatocellular carcinoma (7.84%), colorectal cancer (5.88%), gastroesophageal cancer (5.88%), Hodgkin lymphoma (3.92%) and others (11.76%). The majority of patients had stage IV cancer (82.35%). Among the ICI therapies, anti-PD-1 inhibitor (72.55%) was most frequently used, followed by anti-PD-1 inhibitor combined with anti-CTLA-4 inhibitor (17.65%), anti-PD-1 inhibitor combined with anti-LAG-3 inhibitor (3.92%), and anti-PD-L1 inhibitor (5.88%). Notably, monotherapy with nivolumab or pembrolizumab was the primary ICI therapy, with the maximum average dose of 240 mg and 600 mg, respectively, and the cumulative average cycle of 2.08 and 3.50, respectively. Additionally, 50.98% of patients received concomitant chemotherapy, 23.53% received concomitant radiotherapy, and 13.73% received concomitant tyrosine kinase inhibitor.

**Table 1:** Baseline participant characteristics.

Characteristics	N = 51
Gender	
Male (%)	35 (68.63)
Female (%)	16 (31.37)
Age (year)	60.00 ± 1.99
Female	56.19 ± 3.87
Male	61.74 ± 2.28
Cancer type	
Lung cancer (%)	22 (43.14)
Melanoma (%)	6 (11.76)
Renal cell carcinoma (%)	5 (9.80)
Hepatocellular carcinoma (%)	4 (7.84)
Colorectal cancer (%)	3 (5.88)
Gastroesophageal cancer (%)	3 (5.88)
Hodgkin lymphoma (%)	2 (3.92)
Others (%)	6 (11.76)
Cancer stage	
IV (%)	42 (82.35)
ICI therapy	
Anti-PD-1	37 (72.55)
Anti-PD-1+Anti-CTLA-4	9 (17.65)
Anti-PD-1+Anti-LAG-3	2 (3.92)
Anti-PD-L1	3 (5.88)
Cumulative dose of ICI (mg)	
Nivolumab	240 ± 69.28
Pembrolizumab	600 ± 244.95
Cumulative number of cycles	
Nivolumab	2.08 ± 0.42
Pembrolizumab	3.50 ± 1.06
Concomitant chemotherapy	26 (50.98)
Concomitant radiotherapy	12 (23.53)
Concomitant tyrosine kinase inhibitor	7 (13.73)

To further analyze our findings, we classified the statistical results based on CRS grade, and the results are shown in Table 2, Table 3, Table 4. 27 patients were included in the grade 1–2 CRS group and 24 patients were included in the grade 3–5 CRS group. The baseline characteristics of the two groups were similar, except for the number of patients who received pembrolizumab (Table 2). Of note, the cumulative average dose and cycle of anti-PD-1 inhibitor in the grade 1–2 CRS group were 528.00 mg and 3.00 cycles, respectively, which were higher than 220.00 mg and 2.36 cycles in the grade 3–5 group, but there was no statistical difference (Table 3). Similarly, as the main anti-PD-1 monotherapy in our research, the average duration of nivolumab was 189.83 days in the grade 1–2 CRS group longer than 89.00 days in the grade 3–5 group, while the average duration of pembrolizumab was similar between the two groups. Interestingly, we further found that the average time to CRS was 11.78 days in the grade 1–2 CRS group significantly lower than 30.67 days in the grade 3–5 group, whereas the duration of CRS did not differ between the two groups (Table 3).

**Table 2:** The baseline characteristics of patients with different grades of CRS.

	Grade 1–2 (n = 27)	Grade 3–5 (n = 24)	P
Gender			
Female (%)	9 (33.33)	7 (29.17)	0.772
Male (%)	18 (66.67)	17 (70.83)	0.772
Age (year)	59.04 ± 2.78	61.08 ± 2.90	0.6129
Cancer type			
Lung cancer (%)	11 (40.74)	11 (45.83)	0.782
Melanoma (%)	3 (11.11)	3 (12.50)	1.000
Renal cell carcinoma (%)	3 (11.11)	2 (8.33)	1.000
Hepatocellular carcinoma (%)	2 (7.41)	2 (8.33)	1.000
Colorectal cancer (%)	0 (0.00)	3 (12.50)	0.097
Gastroesophageal cancer (%)	3 (11.11)	0 (0.00)	0.238
hodgkin lymphoma (%)	2 (7.41)	0 (0.00)	0.492
Others (%)	3 (11.11)	3 (12.50)	1.000
Cancer stage			
IV (%)	22 (81.48)	20 (83.33)	1.000
ICI therapy			
Nivolumab	10 (37.04)	7 (29.17)	0.767
Pembrolizumab	13 (48.15)	4 (16.67)	0.021
Nivolumab + Ipilimumab	2 (7.41)	7 (29.17)	0.066
Sintilimab	1 (3.70)	2 (8.33)	0.595
Durvalumab	1 (3.70)	0 (0.00)	1.000
Penpulimab	0 (0.00)	1 (4.17)	0.471
Atezolimumab	0 (0.00)	2 (8.33)	0.216
Avelumab	0 (0.00)	1 (4.17)	0.471
Others	1 (3.70)	1 (4.17)	1.000
Concomitant chemotherapy	13 (48.15)	13 (54.17)	0.781
Concomitant radiotherapy	6 (22.22)	6 (25.00)	1.000
Concomitant tyrosine kinase inhibitor	5 (18.52)	2 (8.33)	0.425

**Table 3:** The relationship between CRS grade and related factors.

	Grade 1–2 (n = 27)	Grade 3–5 (n = 24)	P
Cumulative dose of Anti-PD-1	528.00 ± 202.94	220.00 ± 50.33	0.2301
Cumulative number of Anti-PD-1 cycles	3.00 ± 1.05	2.36 ± 0.37	0.4953
Duration of ICI (Days)			
Nivolumab	189.83 ± 78.65	89.00 ± 46.07	0.4249
Pembrolizumab	126.43 ± 64.60	129.33 ± 72.16	0.9797
Time to CRS (days)	11.78 ± 5.09	30.67 ± 6.05	0.0217
Duration of CRS (days)	14.40 ± 3.93	13.13 ± 2.90	0.8246
CRS toxicities by organ system			
Dermatological	8 (29.63)	8 (33.33)	1.000
Cardiovascular	11 (40.74)	22 (91.67)	＜0.001
Respiratory	5 (18.52)	15 (62.50)	0.002
Neurological	2 (7.41)	11 (45.83)	0.003
Hepatic	9 (33.33)	8 (33.33)	1.000
Renal	6 (22.22)	11 (45.83)	0.136
Gastrointestinal	4 (14.81)	2 (8.33)	0.671
Coagulation	1 (3.70)	7 (29.17)	0.019
Musculoskeletal	0 (0.00)	1 (4.17)	0.471
Endocrinic	1 (3.70)	0 (0.00)	1.000
ICU admission (%)	0 (0.00)	17 (70.83)	＜0.001
Oxygen supplementation	4 (14.81)	20 (83.33)	＜0.001
Hypotension	9 (33.33)	21 (87.50)	＜0.001
High-dose vasopressors used	0 (0.00)	15 (62.50)	＜0.001
Intubation (%)	0 (0.00)	13 (54.17)	＜0.001
Antibiotics	3 (11.11)	11 (45.83)	0.011
Positive microbiological cultures	0 (0.00)	0 (0.00)	
Immunosuppressive therapy			
Glucocorticoid therapy	20 (74.07)	22 (91.67)	0.147
mPSL pulse therapy	1 (3.70)	8 (33.33)	0.008
MMF therapy	1 (3.70)	4 (16.67)	0.183
Intervention except for immunosuppression			
Tocilizumab	1 (3.70)	14 (58.33)	＜0.001
Infliximab	0 (0.00)	1 (4.17)	0.471
IVIg	2 (7.41)	2 (8.33)	1.000
PE	0 (0.00)	1 (4.17)	0.471
CHDF/HD	0 (0.00)	4 (16.67)	0.043
Death	0 (0.00)	5 (20.83)	0.018

**Table 4:** The relationship between CRS grade and inflammatory indicators.

	Grade 1–2 (n = 27)	Grade 3–5 (n = 24)	
WBC(G/L)	11.82 ± 1.96	11.46 ± 2.70	0.9130
Plt (G/L)	309.94 ± 52.25	153.82 ± 36.06	0.0206
Ferritin (ng/mL)	1251.00 ± 173.00	74351.97 ± 51677.42	0.4679
CRP (mg/L)	140.20 ± 18.98	150.03 ± 24.69	0.7526
IL-6 (pg/ml)	42.62 ± 9.94	1271.17 ± 610.97	0.0491
IL-10 (pg/ml)	17.87 ± 7.94	144.81 ± 104.21	0.5550
TNF-α (pg/ml)	26.67 ± 10.67	234.78 ± 184.13	0.5605
IFN-γ(ng/L)	164.80 ± 147.90	2229.57 ± 1442.47	0.4630

Regarding CRS-related toxicity in various organ systems, our analysis revealed that ICI-induced CRS involved multiple systems including the dermatological, cardiovascular, respiratory, neurological, hepatic, renal, gastrointestinal, coagulation, musculoskeletal, and endocrine (Table 3). In particular, patients in the grade 3–5 CRS group exhibited a greater degree of cardiovascular, pulmonary, neurological, and coagulation involvement compared to those in the grade 1–2 CRS group. It is worth noting that ICU admission, oxygen supplementation, hypotension, high-dose vasopressors usage, and intubation were more common in the grade 3–5 CRS group compared with those in the grade 1–2 CRS group. Although there were no positive results in microbiological cultures between the two groups, prophylactic antibiotic use was more prevalent in the grade 3–4 CRS group. Additionally, pulse-dose mPSL, tocilizumab, and continuous hemodiafiltration/hemodialysis (CHDF/HD) were used more frequently in the grade 3–5 CRS group compared to those in the grade 1–2 CRS group, while there was no statistical difference between the two groups in the use of infliximab, intravenous immunoglobulin (IVIg) or plasma exchange (PE). As expected, 20.83% of patients in the grade 3–4 CRS group succumbed to the illness, while no patients in the grade 1–2 CRS group experienced fatalities.

Regarding laboratory tests for ICI-induced CRS, analysis of markers of inflammation revealed that the level of IL-6 was significantly higher in the grade 3–5 CRS group compared to the grade 1–2 CRS group, whereas the platelet count was significantly lower in the former compared to the latter (Table 4). However, no significant differences were observed between the two groups in terms of white cell count, ferritin level, CRP level, or levels of IL-10, TNF-α, or interferon (IFN)-γ.

## 4 Discussion

CRS is a systemic inflammatory response that is characterized by the release of a variety of cytokines, such as INF-γ, TNF-α, IL-1β, IL-2, and IL-6 caused by an overshooting immune response mediated by T cells, B cells, NK cells, and macrophages [31]. CRS can be triggered by a variety of factors. Depending on the cause, CRS can be classified as drug-induced CRS and infection-induced CRS [32]. Several antibody-based therapies such as the CD28 superagonist TGN1412 [33], rituximab [34], obinutuzumab [35], alemtuzumab [36], and nivolumab [8], as well as non-protein-based cancer drugs, such as oxaliplatin [37] and lenalidomide [38], have been shown to cause CRS. Massive T cell stimulation has also been proposed as a cytokine storm in the context of severe viral infections such as influenza [39,40]. Lately, with the successful use of new T-cell immunotherapy drugs in solid tumors, leukemias, and lymphomas has led to an increase in the incidence of CRS [30]. As an irAE, CRS can be a severe and life-threatening complication. Currently, there are limited reports on ICI-induced CRS, and there is a lack of uniform diagnostic criteria or treatment guidelines. Due to the release of multiple inflammatory cytokines, the most accepted diagnostic criteria on CRS are a constellation of inflammatory symptoms such as fever, nausea, headache, tachycardia, hypotension, rash, and shortness of breath [according to the CTCAE version 4.0 (National Cancer Institute Common Terminology Criteria for Adverse Events)]. And the CRS was evidenced by raised inflammatory markers, thrombocytopenia, elevated cytokine levels and steroid responsiveness [41]. Symptoms appear suddenly after exposure to the triggering factor. Diagnosis remains primarily clinical, based on scientific recommendations after the exclusion of differential diagnoses. In this study, we present two severe cases of ICI-induced CRS after the first intravenous dose of anti-PD-1 inhibitor and provide a summary and analysis of ICI-induced CRS case series to explore the possible causes of CRS and to provide guidance for therapy. Our analysis revealed that ICI-induced CRS was more common in male patients with advanced cancers, consistent with previous study [12]. In addition, study had shown that male sex appeared to be a risk factor for some irAEs [42]. However, the reason for the association between gender and CRS remains unclear. Since the majority of cancer types in our study were lung cancers, particularly NSCLC, we speculate that the higher susceptibility of men to NSCLC may be a contributing factor.

Although treatment duration, drug interactions, disease burden, baseline cytokine levels, and cancer types have been shown to play an important role in irAEs [4,22,[43], [44], [45]], there is no clear association between ICIs dose and toxicity, particularly in solid tumors [44]. Whether the factors mentioned above are related to the severity of CRS have not been directly reported. Our study showed that the cumulative dose and cycle of anti-PD-1 inhibitor, or even the duration of nivolumab and pembrolizumab, had no difference between the patients with grade 1–2 CRS and grade 3–5 CRS. However, these results are not conclusive due to the large standard error in our study. Further research with a larger sample size is necessary. Previous study has shown CRS can occur immediately, minutes, hours, days, or weeks after the administration of therapy [46]. In our study, the average total time to CRS was 33.69 days, and patients with grade 3–5 CRS had significantly longer time to onset of CRS compared to grade 1–2 CRS. A recent study defined the time to fever as the time to CRS and also demonstrated that the median time to fever in patients with grade 3–4 CRS was longer than that in patients with grade 1–2 CRS [12], indicating that the longer time to CRS may be related to the severity of CRS.

Patients with CRS can exhibit a broad spectrum of symptoms, beginning with a fever, and may result in severe multi-organ failure, shock, and disseminated intravascular coagulation due to a systemic inflammatory response [47,48]. Our study found that CRS can impact virtually all organs within the human body. Notably, the incidence of cardiovascular, respiratory, neurological, and coagulation toxicity in grade 3–5 CRS was higher than that of grade 1–2 CRS, indicating a correlation between the severity of CRS and these types of toxicity. Furthermore, patients with advanced grade CRS had higher rates of ICU admission, oxygen supplementation, hypotension, high-dose vasopressors usage, and intubation. Cardiovascular and respiratory toxicity is relatively common in ICI treatments and related to the high mortality [3]. Hypotension and capillary leak, often resulting in hypoxia and pulmonary edema, are distinguishing characteristics of severe CRS [49]. Moreover, organ dysfunction may arise as a secondary effect of hypotension, hypoxia, or massive cytokines release [50,51]. Encephalitis was the primary neurological toxicity in our study. The incidence of ICI-induced encephalitis is estimated to be less than 1%, yet it has the potential to be fatal or to cause lasting (sometimes permanent) impairment of neurological function [[52], [53], [54]]. Furthermore, despite the sequential organ failure assessment (SOFA) score being developed for sepsis, recent studies have suggested that the SOFA score plays a significant role in predicting prognosis in oncology patients with systemic inflammatory syndrome, independent of infection. SOFA score greater than 2 are associated with mortalities ranging from 27.9% to 47.8% [55,56], suggesting that more extensive organ damage is associated with more severe CRS.

CRS is primarily characterized by immune hyperactivation, necessitating the exclusion of infection for an accurate diagnosis, particularly given the immunosuppressed status of most patients, which increases their susceptibility to sepsis or neutropenic fever. In cases of severe CRS, empirical administration of antibiotics is frequently necessary. The pathology of CRS is mediated by various cytokines, including IL-6, TNF-α, IFN-γ, IL-2, IL-8, IL-10, and granulocyte-macrophage colony-stimulating factor (GM-CSF), among which IL-6 has been implicated as playing a pivotal role in the immunopathogenesis of CRS [57]. It has been shown that genetic variants in the *il6* gene can result in over-expression of IL-6 via *trans*-signaling pathway, and that polymorphisms in this gene may predispose patients to ICI-induced CRS [6]. High levels of IL-6 have been observed in patients with severe CRS, with such levels being attributed to T cell proliferation resulting from therapeutic and inflammatory processes [57]. Our study revealed that patients with grade 3–5 CRS exhibited significantly elevated levels of IL-6 and markedly reduced platelet count, indicating a possible relationship between the severity of CRS and the levels of IL-6 and platelet counts. The lower platelet count is considered to cause higher grade CRS due to macrophage activation [12]. Furthermore, while the levels of TNF-α, IFN-γ, IL-10, and CRP in patients with grade 3–5 CRS were higher than those in patients with grade 1–2 CRS, no significant difference was noted between the two groups, possibly due to a lack of sufficient data.

Managing ICI-induced CRS can be a daunting task. As per the present study, CRS is a non-antigen-specific toxicity [58], hence its management may different from other irAEs. Severity and underlying causes are crucial factors in CRS management. Supportive care and vigilant monitoring are recommended for mild CRS cases. For patients with mild to moderate symptoms, the first-line treatment is the administration of intravenous mPSL [59]. Sever CRS may necessitate ICU admission due to common occurrences of hypotension and hypoxia. In such cases, mPSL pulse therapy is recommend, and glucocorticoids are generally tapered rapidly after 3–5 days, considering the potential complications in nonhematologic malignancies. Additionally, several cytokine antagonists have been described as potential therapeutic candidates. Tocilizumab (the IL-6 inhibitor) is recommended for all patients experiencing more than grade 2 CRS, and to patients with more than grade 1 CRS with comorbidities or elderly [57]. Fever and hypotension can be resolved within a few hours for patients who respond to tocilizumab, allowing for the quick weaning of vasopressors and other supportive measures [57]. In our case series, 15 patients received tocilizumab, with only one patient experiencing life-threatening toxicity eventually. Other cytokine-targeted therapies such as etanercept (TNF-α inhibitor), dalclizumab (IL-2R antagonist) and anakinra (IL-1R antagonist), as well as IVIg and PE, may have a potential role in managing the syndrome [30,60,61]. However, their efficacy, indications and limitations have yet to be evaluated. In summary, measuring cytokine levels, particularly in higher grades of CRS, may be advantageous in adjusting management regimens.

## 5 Conclusion

ICI-induced CRS is a rare yet perilous complication. The initial symptoms of CRS range from mild to severe multiorgan dysfunction. Patients with longer time to CRS onset exhibit a higher level of IL-6 and lower platelet count, enabling us to predict those likely to develop severe CRS. Higher-grade CRS is associated with cardiovascular, respiratory, neurological, and coagulation toxicities, which serve as markers for patients requiring ICU admission, oxygen supplementation, hypotension, high-dose vasopressors usage, and intubation. Our study underscores the importance of recognizing CRS as a leading cause of severe multiorgan dysfunction following the initiation of ICI therapy. Therefore, further research should be undertaken to prevent and manage CRS.

## Ethics statement

Informed consent was obtained from the patient (or relatives) for publication of all images and clinical data in this case report.

## Funding

This work was supported by the 10.13039/501100012166National Key Research and Development Program of China (2021YFC2501804).

## Data availability statement

The data are available from the corresponding author.

## CRediT authorship contribution statement

**Yujing Zhang:** Writing – original draft, Project administration, Methodology, Data curation, Conceptualization. **Xiaoyue Wen:** Methodology, Data curation. **Yaqi OuYang:** Methodology, Data curation. **Yingying Hu:** Supervision, Software. **Xiangzhi Fang:** Validation, Data curation. **Jiancheng Zhang:** Writing – review & editing, Project administration. **Yin Yuan:** Writing – review & editing, Project administration.

## Declaration of competing interest

The authors declare that they have no known competing financial interests or personal relationships that could have appeared to influence the work reported in this paper.

## Figures and Tables

**figs1:**
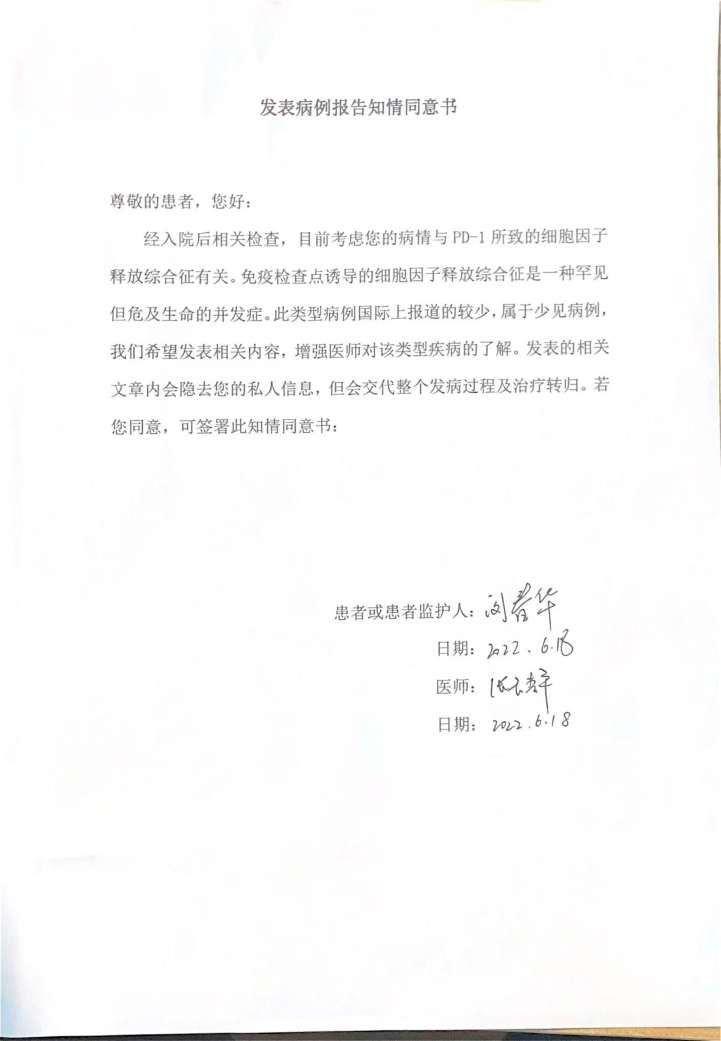


**figs2:**
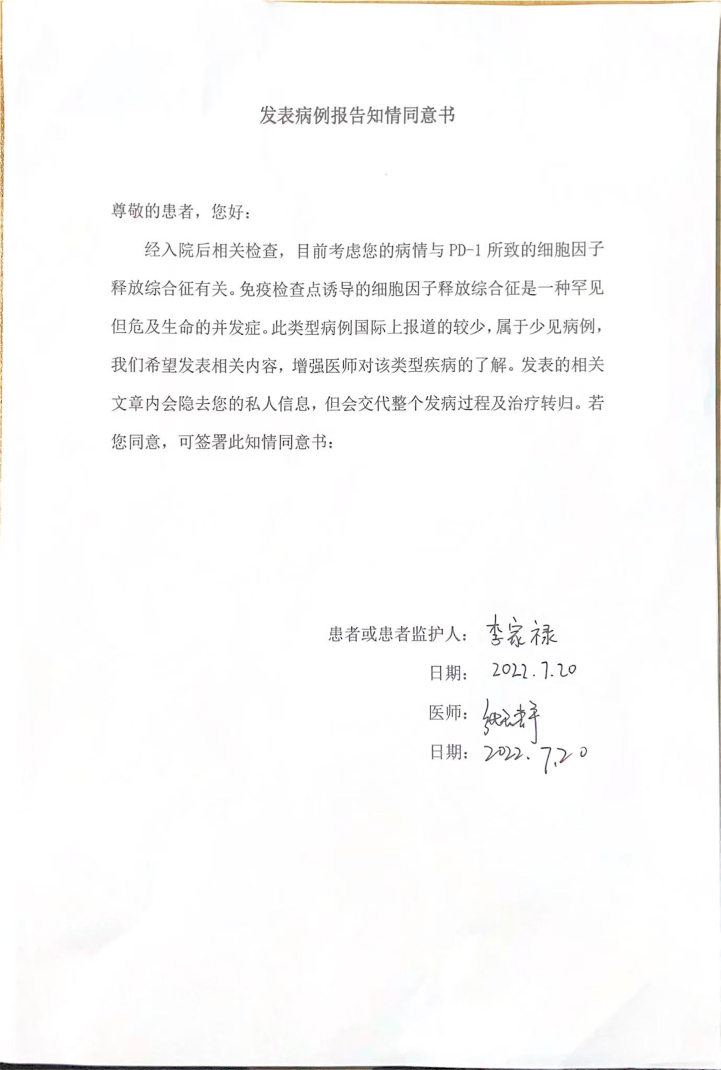


## Abbreviations

ALT: alanine aminotransferase
AST: aspartate aminotransferase
CHDF/HD: continuous hemodiafiltration/hemodialysis
CRP: C-reactive protein
CRS: Cytokine release syndrome
CTLA-4: anti-cytotoxic T lymphocyte associated antigen-4
ICIs: Immune checkpoint inhibitor
ICU: intensive care unit
IL: interleukin
IFN: interferon
irAEs: Immune-related adverse events
IVIg: immunoglobulin
G-CSF: granulocyte colony-stimulating factor
HBV: hepatitis B virus
mPSL: methylprednisolone
NSCLC: non-small cell lung cancer
PD-1: programmed cell death receptor-1
PD-L1: PD-1 ligand 1
PE: plasma exchange
PET/CT: positron emission tomography/computed tomography
TNF: tumor necrosis factor

## References

[1] Hargadon K.M., Johnson C.E., Williams C.J. (2018). Immune checkpoint blockade therapy for cancer: an overview of FDA-approved immune checkpoint inhibitors. Int. Immunopharm..

[2] Das S., Johnson D.B. (2019). Immune-related adverse events and anti-tumor efficacy of immune checkpoint inhibitors. J. Immunother. Cancer.

[3] Brahmer J.R., Abu-Sbeih H., Ascierto P.A., Brufsky J., Cappelli L.C., Cortazar F.B. (2021). Society for Immunotherapy of Cancer (SITC) clinical practice guideline on immune checkpoint inhibitor-related adverse events. J. Immunother. Cancer.

[4] Arnaud-Coffin P., Maillet D., Gan H.K., Stelmes J.J., You B., Dalle S., Péron J. (2019). A systematic review of adverse events in randomized trials assessing immune checkpoint inhibitors. Int. J. Cancer.

[5] Chatenoud L., Ferran C., Reuter A., Legendre C., Gevaert Y., Kreis H. (1989). Systemic reaction to the anti-T-cell monoclonal antibody OKT3 in relation to serum levels of tumor necrosis factor and interferon-α. N. Engl. J. Med..

[6] Ceschi A., Noseda R., Palin K., Verhamme K. (2020). Immune checkpoint inhibitor-related cytokine release syndrome: analysis of WHO global pharmacovigilance database. Front. Pharmacol..

[7] Urasaki T., Ono M., Mochizuki T., Takeda K., Nishizawa A., Fukagawa E. (2021). Case report: a case of trimethoprim/sulfamethoxazole-triggered hypotensive shock: cytokine release syndrome related to immune checkpoint inhibitors and drug-induced hypersensitivity syndrome. Front. Oncol..

[8] Rotz S.J., Leino D., Szabo S., Mangino J.L., Turpin B.K., Pressey J.G. (2017). Severe cytokine release syndrome in a patient receiving PD-1-directed therapy. Pediatr. Blood Cancer.

[9] Oda H., Ishihara M., Miyahara Y., Nakamura J., Kozuka Y., Iwasa M. (2019). First case of cytokine release syndrome after nivolumab for gastric cancer. Case Rep. Oncol..

[10] Honjo O., Kubo T., Sugaya F., Nishizaka T., Kato K., Hirohashi Y. (2019). Severe cytokine release syndrome resulting in purpura fulminans despite successful response to nivolumab therapy in a patient with pleomorphic carcinoma of the lung: a case report. J. Immunother. Cancer.

[11] Adashek M.L., Feldman M. (2019). Cytokine release syndrome resulting from anti-programmed death-1 antibody: raising awareness among community oncologists. J. Oncol. Pract..

[12] Tay S.H., Toh M.M.X., Thian Y.L., Vellayappan B.A., Fairhurst A.M., Chan Y.H. (2022). Cytokine release syndrome in cancer patients receiving immune checkpoint inhibitors: a case series of 25 patients and review of the literature. Front. Immunol..

[13] Dimitriou F., Matter A.V., Mangana J., Urosevic-Maiwald M., Micaletto S., Braun R.P. (2019). Cytokine release syndrome during sequential treatment with immune checkpoint inhibitors and kinase inhibitors for metastatic melanoma. J. Immunother..

[14] Yomota M., Mirokuji K., Sakaguchi M., Kitahara Y., Chin F., Setoguchi K., Hosomi Y. (2021). Cytokine release syndrome induced by immune-checkpoint inhibitor therapy for non-small-cell lung cancer. Intern. Med..

[15] Amlani A., Barber C., Fifi-Mah A., Monzon J. (2020). Successful treatment of cytokine release syndrome with IL-6 blockade in a patient transitioning from immune-checkpoint to MEK/BRAF inhibition: a case report and review of literature. Oncol..

[16] Ohira J., Kawamoto M., Sugino Y., Kohara N. (2020). A case report of fulminant cytokine release syndrome complicated by dermatomyositis after the combination therapy with immune checkpoint inhibitors. Medicine (Baltim.).

[17] Zhao L., Yang Y., Li W., Li T., Gao Q. (2018). Nivolumab-induced cytokine-release syndrome in relapsed/refractory Hodgkin's lymphoma: a case report and literature review. Immunotherapy.

[18] Ciner A.T., Hochster H.S., August D.A., Carpizo D.R., Spencer K.R. (2021). Delayed cytokine release syndrome after neoadjuvant nivolumab: a case report and literature review. Immunotherapy.

[19] Murata D., Azuma K., Tokisawa S., Tokito T., Hoshino T. (2022). A case of cytokine release syndrome accompanied with COVID-19 infection during treatment with immune checkpoint inhibitors for non-small cell lung cancer. Thorac. Cancer.

[20] Kunimasa K., Inoue T., Matsueda K., Kawamura T., Tamiya M., Nishino K., Kumagai T. (2022). Cytokine release syndrome and immune-related pneumonitis associated with tumor progression in a pulmonary pleomorphic carcinoma treated with nivolumab plus ipilimumab treatment: a case report. JTO Clin. Res. Rep..

[21] Sackstein P., Zaemes J., Kim C. (2021). Pembrolizumab-induced cytokine release syndrome in a patient with metastatic lung adenocarcinoma: a case report. J. Immunother. Cancer.

[22] Zhang M., Cheng Y., Hu Y., Nie L. (2022). Cytokine release syndrome and successful response to pembrolizumab therapy in a patient with EGFR-mutated non-small-cell lung cancer: a case report. Thorac Cancer.

[23] Deng P.B., Jiang J., Hu C.P., Cao L.M., Li M. (2022). Tumor-related cytokine release syndrome in a treatment-naïve patient with lung adenocarcinoma: a case report. World J. Clin. Cases.

[24] Normand C.V., Zender H.O., Staehli D.M., Chouiter-Djebaili A.F., John G. (2021). Acute cytokine release syndrome after a first dose of pembrolizumab as second-line treatment for metastatic, programmed death-ligand 1-positive, non-small-cell lung cancer. J. Oncol. Pharm. Pract..

[25] Rassy E.E., Assi T., Rizkallah J., Kattan J. (2017). Diffuse edema suggestive of cytokine release syndrome in a metastatic lung carcinoma patient treated with pembrolizumab. Immunotherapy.

[26] Kogure Y., Ishii Y., Oki M. (2019). Cytokine release syndrome with pseudoprogression in a patient with advanced non-small cell lung cancer treated with pembrolizumab. J. Thorac. Oncol..

[27] Hu J., Li Y., Chen X., Luo C., Zuo X. (2020). Pulmonary fibrosis and cytokine release syndrome after hyperactivation with sintilimab. J. Clin. Pharm. Therapeut..

[28] Gao C., Xu J., Han C., Wang L., Zhou W., Yu Q. (2020). An esophageal cancer case of cytokine release syndrome with multiple-organ injury induced by an anti-PD-1 drug: a case report. Ann. Palliat. Med..

[29] Sindel A., Taylor T., Chesney A., Clark W., Fowler A.A., Toor A.A. (2019). Hematopoietic stem cell mobilization following PD-1 blockade: cytokine release syndrome after transplantation managed with ascorbic acid. Eur. J. Haematol..

[30] Menakuru S.R., Azeem Q., Priscu A., Khan I., Beirat A. (2022). Stage 4 cytokine release syndrome caused by the first dose of nivolumab and ipilimumab combination therapy in a patient with metastatic melanoma successfully treated with methylprednisolone, tocilizumab, and etanercept. Case Rep. Oncol..

[31] D.W. Lee, R. Gardner, D.L.J.B. Porter, Current concepts in the diagnosis and management of cytokine release syndrome, Blood, 126(8), 1048-1048. https://doi:10.1182/blood-2015-07-656918.. 10.1182/blood-2014-05-552729 PMC4093680 24876563

[32] Shimabukuro-Vornhagen A., Gödel P., Subklewe M., Stemmler H.J., Schlößer H.A., Schlaak M. (2018). Cytokine release syndrome. J. Immunother. Cancer.

[33] Suntharalingam G., Perry M.R., Ward S., Brett S.J., Castello-Cortes A., Brunner M.D., Panoskaltsis N. (2006). Cytokine storm in a phase 1 trial of the anti-CD28 monoclonal antibody TGN1412. N. Engl. J. Med..

[34] Winkler U., Jensen M., Manzke O., Schulz H., Diehl V., Engert A. (1999). Cytokine-release syndrome in patients with B-cell chronic lymphocytic leukemia and high lymphocyte counts after treatment with an anti-CD20 monoclonal antibody (rituximab, IDEC-C2B8). Blood.

[35] Freeman C.L., Morschhauser F., Sehn L., Dixon M., Houghton R., Lamy T. (2015). Cytokine release in patients with CLL treated with obinutuzumab and possible relationship with infusion-related reactions. Blood.

[36] Wing M.G., Moreau T., Greenwood J., Smith R.M., Hale G., Isaacs J. (1996). Mechanism of first-dose cytokine-release syndrome by CAMPATH 1-H: involvement of CD16 (FcgammaRIII) and CD11a/CD18 (LFA-1) on NK cells. J. Clin. Invest..

[37] Tonini G., Santini D., Vincenzi B., Borzomati D., Dicuonzo G., La Cesa A. (2002). Oxaliplatin may induce cytokine-release syndrome in colorectal cancer patients. J. Biol. Regul. Homeost. Agents.

[38] Aue G., Njuguna N., Tian X., Soto S., Hughes T., Vire B. (2009). Lenalidomide-induced upregulation of CD80 on tumor cells correlates with T-cell activation, the rapid onset of a cytokine release syndrome and leukemic cell clearance in chronic lymphocytic leukemia. Haematologica.

[39] Tisoncik J.R., Korth M.J., Simmons C.P., Farrar J., Martin T.R., Katze M.G. (2012). Into the eye of the cytokine storm. Microbiol. Mol. Biol. Rev..

[40] de Jong M.D., Simmons C.P., Thanh T.T., Hien V.M., Smith G.J., Chau T.N. (2006). Fatal outcome of human influenza A (H5N1) is associated with high viral load and hypercytokinemia. Nat. Med..

[41] Au L., Fendler A., Shepherd S.T.C., Rzeniewicz K., Cerrone M., Byrne F. (2021). Cytokine release syndrome in a patient with colorectal cancer after vaccination with BNT162b2. Nat. Med..

[42] Faje A.T., Sullivan R., Lawrence D., Tritos N.A., Fadden R., Klibanski A., Nachtigall L. (2014). Ipilimumab-induced hypophysitis: a detailed longitudinal analysis in a large cohort of patients with metastatic melanoma. J. Clin. Endocrinol. Metab..

[43] Mok T.S.K., Wu Y.L., Kudaba I., Kowalski D.M., Cho B.C., Turna H.Z. (2019). Pembrolizumab versus chemotherapy for previously untreated, PD-L1-expressing, locally advanced or metastatic non-small-cell lung cancer (KEYNOTE-042): a randomised, open-label, controlled, phase 3 trial. Lancet.

[44] Shimizu T., Seto T., Hirai F., Takenoyama M., Nosaki K., Tsurutani J. (2016). Phase 1 study of pembrolizumab (MK-3475; anti-PD-1 monoclonal antibody) in Japanese patients with advanced solid tumors. Invest. N. Drugs.

[45] Wang P.F., Chen Y., Song S.Y., Wang T.J., Ji W.J., Li S.W. (2017). Immune-related adverse events associated with anti-PD-1/PD-L1 treatment for malignancies: a meta-analysis. Front. Pharmacol..

[46] Santomasso B.D., Nastoupil L.J., Adkins S., Lacchetti C., Schneider B.J., Anadkat M. (2021). Management of immune-related adverse events in patients treated with chimeric antigen receptor T-cell therapy: ASCO guideline. J. Clin. Oncol..

[47] Makarious D., Horwood K., Coward J.I.G. (2017). Myasthenia gravis: an emerging toxicity of immune checkpoint inhibitors. Eur. J. Cancer.

[48] Cho J., Ahn M.-J., Yoo K., Lee H., Kim H., Heo M. (2017). A phase II study of pembrolizumab for patients with previously treated advanced thymic epithelial tumor. J. Clin. Oncol..

[49] Porter D., Frey N., Wood P.A., Weng Y., Grupp S.A. (2018). Grading of cytokine release syndrome associated with the CAR T cell therapy tisagenlecleucel. J. Hematol. Oncol..

[50] Morris E.C., Neelapu S.S., Giavridis T., Sadelain M. (2022). Cytokine release syndrome and associated neurotoxicity in cancer immunotherapy. Nat. Rev. Immunol..

[51] Wang Z., Han W. (2018). Biomarkers of cytokine release syndrome and neurotoxicity related to CAR-T cell therapy. Biomark. Res..

[52] Johnson D.B., Manouchehri A., Haugh A.M., Quach H.T., Balko J.M., Lebrun-Vignes B. (2019). Neurologic toxicity associated with immune checkpoint inhibitors: a pharmacovigilance study. J. Immunother. Cancer.

[53] Spain L., Walls G., Julve M., O'Meara K., Schmid T., Kalaitzaki E. (2017). Neurotoxicity from immune-checkpoint inhibition in the treatment of melanoma: a single centre experience and review of the literature. Ann. Oncol..

[54] Conry R. (2016). https://doi:10.29245/2572.942X/2016/4.1040.

[55] Probst L., Schalk E., Liebregts T., Zeremski V., Tzalavras A., von Bergwelt-Baildon M. (2019). Prognostic accuracy of SOFA, qSOFA and SIRS criteria in hematological cancer patients: a retrospective multicenter study. J Intensive Care.

[56] Chae B.R., Kim Y.J., Lee Y.S. (2020). Prognostic accuracy of the sequential organ failure assessment (SOFA) and quick SOFA for mortality in cancer patients with sepsis defined by systemic inflammatory response syndrome (SIRS). Support. Care Cancer.

[57] Lee D.W., Gardner R., Porter D.L., Louis C.U., Ahmed N., Jensen M. (2014). Current concepts in the diagnosis and management of cytokine release syndrome. Blood.

[58] Si S., Teachey D.T. (2020). Spotlight on tocilizumab in the treatment of CAR-T-cell-induced cytokine release syndrome: clinical evidence to date. Therapeut. Clin. Risk Manag..

[59] Aldea M., Orillard E., Mansi L., Marabelle A., Scotte F., Lambotte O., Michot J.M. (2020). How to manage patients with corticosteroids in oncology in the era of immunotherapy?. Eur. J. Cancer.

[60] Vessillier S., Eastwood D., Fox B., Sathish J., Sethu S., Dougall T. (2015). Cytokine release assays for the prediction of therapeutic mAb safety in first-in man trials--Whole blood cytokine release assays are poorly predictive for TGN1412 cytokine storm. J. Immunol. Methods.

[61] Giavridis T., van der Stegen S.J.C., Eyquem J., Hamieh M., Piersigilli A., Sadelain M. (2018). CAR T cell-induced cytokine release syndrome is mediated by macrophages and abated by IL-1 blockade. Nat. Med..

