# Supplementary material for: Severe cytokine release syndrome induced by immune checkpoint inhibitors in cancer patients – A case report and review of the literature
Source: Heliyon. 2024 Jan 10;10(2):e24380. doi: 10.1016/j.heliyon.2024.e24380 (PMC10826737; doi:10.1016/j.heliyon.2024.e24380)
Supplement: Multimedia component 1 [file mmc1.docx]

**Supplementary Data. the basic information in patients with ICI-induced CRS**

| Ref | Patient No. | Gender / Age (year) | Tumor / stage | Medical history | ICI therapy | Concomitant other therapy | Clinical symptoms | Immunosuppressive therapy | Other interventions | Grade of CRS | Time to CRS onset (from first administration) (days) | Duration of CRS (days) | Lab tests | Outcome |
| --- | --- | --- | --- | --- | --- | --- | --- | --- | --- | --- | --- | --- | --- | --- |
| Urasaki T 2021 [7] | 1 | F / 46 | Metastatic pulmonary tumors / IV | Renal cell carcinoma | Nivolumab (3 mg/kg) and ipilimumab (1 mg/kg); 4 cycles | - | Fever, hypotension, respiratory failure, shock, platelet decreased, liver and renal failure, coagulopathy, rash. | HDC (300 mg/day), mPSL pulse therapy (500 mg/day, for 3 days), PSL (50 mg/day); tocilizumab (8mg/kg); IVIg (20g/d, for 5days) | ICU admission, oxygen supplementation, intubation, HD, PE  high-dose vasopressors used | 4 | 53 | 12 | N.A. | Recovered |
| Rotz et al. 2017 [8] | 2 | F / 29 | Alveolar soft part sarcoma / IV | - | Nivolumab (3 mg/kg); 2 cycles | Radiotherapy and tyrosine kinase inhibitor | Fever, tachycardia, rash, encephalopathy, hypotension, hypoxia, hepatic dysfunction, acute kidney injury, and coagulopathy. | HDC (50 mg/m^2^/day), mPSL (2 mg/kg/day); tocilizumab (1st.: 4mg/kg, 2nd.: 8 mg/kg) | Oxygen supplementation | 3 | 18 | 7 | PLT 127 × 10^9^/L, IL-6 45 pg/mL, IL-8 125 pg/mL, IL-10 128 pg/mL, IFN-γ 660 pg/mL, CRP 4.97 mg/L | Recovered |
| Oda et al. 2019 [9] | 3 | M / 43 | Gastric cancer / IV | - | Nivolumab; 1 cycle | Chemotherapy and tyrosine kinase inhibitor | Fever, tachycardia, appetite loss, malaise, hepatic dysfunction. | PSL (80 mg/day), mPSL pulse therapy (1g/day, for 3 days); MMF (2000 mg/day) | Not particular | 2 | 8 | 30 | WBC 6.03 × 10^9^/L, PLT 101 × 10^9^/L, CRP 64 mg/L | Progressive disease |
| Honjo et al. 2019 [10] | 4 | F / 52 | Lung cancer / IIIb | - | Nivolumab (120 mg per cycle); 1 cycle | Chemotherapy | Asthenia, fever, dermatological toxicity, hepatic dysfunction, muscle soreness, cardiac hypofunction, respiratory distress, renal failure, gangrene in the lower extremities | mPSL pulse therapy (1g/day, for 3 days), PSL (50 mg/day); MMF | ICU admission, oxygen supplementation, intubation, high-dose vasopressors used, CHDF | 4 | 14 | 7 | PLT 11 × 10^9^/L, CRP 417.9 mg/L, Ferritin 3877 ng/mL, IL-1β 34.9 pg/mL, IL-2 < 15.6 pg/mL, soluble IL-2R 3630 U/mL, IL-6 1510 pg/mL, IL-10 20.0 pg/mL, IL-12 <0.78 pg/mL, TNFα 251 pg/mL, IFN-γ 9260 ng/L, G-CSF 753 pg/mL | Recovered |
| Adashek et al. 2019 [11] | 5 | M / 72 | NSCLC / IV | - | Pembrolizumab（4 cycles） | Chemotherapy and radiotherapy | Hypotension, respiratory distress, encephalopathy | Tocilizumab | ICU admission, oxygen supplementation, intubation; high-dose vasopressors used | 4 | 63 | 1 | N.A. | Recovered |
| Tay et al. 2022 [12] | 6 | M / 44 | Adrenocortical carcinoma / IV | - | Nivolumab; 63 days | Chemotherapy | Fever | - | Not particular | 1 | 0 | N.A. | WBC 6.71 × 10^9^/L, PLT 156 × 10^9^/L, CRP 200 mg/L | Recovered |
|  | 7 | F / 70 | Melanoma / IV | - | Nivolumab; 107 days | Radiotherapy | Fever and transaminitis | PSL (30 mg/day); tocilizumab (8 mg/kg) | Not particular | 1 | 0 | N.A. | WBC 6.79 × 10^9^/L, PLT 328 × 10^9^/L, CRP 117 mg/L, IL-6 19.3 pg/mL | Recovered |
|  | 8 | F / 64 | NSCLC / IV | - | Nivolumab; 28 days | Chemotherapy | Fever, lethargy, dyspnea and transaminitis | PSL (40 mg/day); PSL (60 mg/day, for 3 days) | Oxygen supplementation | 1 | 23 | N.A. | WBC 41.51 × 10^9^/L, PLT 535 × 10^9^/L, CRP 214 mg/L | Recovered |
|  | 9 | M / 64 | Renal cell carcinoma / IV | - | Nivolumab; 275 days | - | Fever | - | Not particular | 1 | 19 | N.A. | WBC 9.54 × 10^9^/L, PLT 612 × 10^9^/L, CRP 182 mg/L | Recovered |
|  | 10 | F / 61 | Melanoma / IV | - | Pembrolizumab,  Nivolumab + ipilimumab; 231 days | - | Fever | PSL (5 mg/day) | Not particular | 1 | 173 | N.A. | WBC 9.01 × 10^9^/L, PLT 333 × 10^9^/L, CRP 83 mg/L, IL-6 63.4 pg/mL | N.A. |
|  | 11 | M / 75 | NSCLC / IV | - | Nivolumab; 543 days | - | Fever | - | Not particular | 1 | 0 | N.A. | WBC 19.74 × 10^9^/L, PLT 566 × 10^9^/L, CRP 213 mg/L | N.A. |
|  | 12 | M / 63 | Renal cell carcinoma / IV | - | Nivolumab + ipilimumab; 21 days | Radiotherapy | Fever, transaminitis | mPSL (2 mg/kg/day, for 3 adys); PSL (60 mg/day); MMF | Not particular | 2 | 0 | N.A. | WBC 12.72 × 10^9^/L, PLT 412 × 10^9^/L, CRP 17 mg/L | Recovered |
|  | 13 | M / 57 | NSCLC / IV | - | Pembrolizumab; 497 days | Chemotherapy | Fever, atrial flutter, dermatitis | PSL (30 mg/day) | Not particular | 2 | 3 | N.A. | WBC 13.87 × 10^9^/L, PLT 180 × 10^9^/L, CRP 265 mg/L | Recovered |
|  | 14 | F / 67 | Endometrial carcinoma / IV | - | Pembrolizumab; 21 days | Chemotherapy, radiotherapy and tyrosine kinase inhibitor | Fever | - | Not particular | 2 | 3 | N.A. | WBC 12.75 × 10^9^/L, PLT 298 × 10^9^/L, CRP 214 mg/L | Recovered |
|  | 15 | M / 70 | NSCLC / IV | - | Pembrolizumab; 21 days | - | Fever, hypotension (BP 83/53 mmHg) | mPSL (2 mg/kg/day, for 3 adys); PSL (60 mg/day) | Not particular | 2 | 1 | N.A. | WBC 11.6 × 10^9^/L, PLT 285 × 10^9^/L, CRP 170 mg/L, IL-6 6.3 pg/mL, Lactate 1.8 mmol/L | Recovered |
|  | 16 | M / 56 | NSCLC / IV | - | Pembrolizumab; 21 days | - | Fever, vomiting, diarrhea | mPSL (2 mg/kg/day, for 3 adys); PSL (60 mg/day) | Not particular | 2 | 0 | N.A. | WBC 9.87 × 10^9^/L, PLT 892 × 10^9^/L, CRP 343 mg/L | Recovered |
|  | 17 | M / 46 | Renal cell carcinoma / IV | - | Nivolumab; 123 days | Radiotherapy and tyrosine kinase inhibitor | Fever, hypotension (BP 80/39 mmHg), acute renal impairment, rash | PSL (40 mg/day); PSL (50 mg/day) | Not particular | 2 | 0 | N.A. | WBC 10.46 × 10^9^/L, PLT 431 × 10^9^/L, CRP 40 mg/L, Lactate 2.5 mmol/L | Recovered |
|  | 18 | M / 76 | Hepatocellular carcinoma / N.A. | - | Durvalumab | Tyrosine kinase inhibitor | Fever, myositis, dermatitis, renal impairment | PSL 30 mg/day | Not particular | 2 | N.A. | N.A. | WBC 11.06 × 10^9^/L, PLT 129 × 10^9^/L, CRP 127 mg/L | N.A. |
|  | 19 | F / 78 | Hepatocellular carcinoma / IV | - | Pembrolizumab; 90 days | Tyrosine kinase inhibitor | Fever, dermatitis, cytopenias, transaminitis | N.A. | Not particular | 2 | 2 | N.A. | WBC 3.38 × 10^9^/L, PLT 129 × 10^9^/L, CRP 70 mg/L, Ferritin 1424 ng/mL, IL-6 37.5 pg/mL | Recovered |
|  | 20 | M / 58 | Esophageal SCC / IV | - | Pembrolizumab; 78 days | Chemotherapy | Fever, hypotension (BP 75/49 mmHg), transaminitis | HDC (300 mg/days); PSL (35 mg/day) | Not particular | 2 | 36 | N.A. | WBC 15.48 × 10^9^/L, PLT 223 × 10^9^/L, CRP 109 mg/L | Recovered |
|  | 21 | M / 70 | NSCLC / IV | - | Pembrolizumab; 157 days | Chemotherapy | Fever, hypotension, diarrhea, dermatitis | HDC (300 mg/days); PSL (50 mg/day) | Not particular | 2 | 12 | N.A. | WBC 3.05 × 10^9^/L, PLT 188 × 10^9^/L, CRP 45 mg/L, Ferritin 1078 ng/mL, IL-6 1.3 pg/mL, IFNγ 16.9 pg/mL | Recovered |
|  | 22 | F / 44 | Breast cancer / IV | - | Anti-PD1 and anti-LAG3; 28 days | Chemotherapy | Fever, diarrhea, dermatitis | - | Not particular | 2 | 3 | N.A. | WBC 3.45 × 10^9^/L, PLT 245 × 10^9^/L, CRP 62 mg/L | N.A. |
|  | 23 | M / 59 | Renal cell carcinoma / IV | - | Nivolumab + ipilimumab; 141 days | - | Fever, hypotension, respiratory failure | mPSL (2 mg/kg/day); PSL (45 mg/day); tocilizumab (8 mg/kg) | ICU admission and oxygen supplementation but did not require intubation | 3 | 195 | N.A. | WBC 10.3 × 10^9^/L, PLT 543 × 10^9^/L, CRP 68 mg/L | Recovered |
|  | 24 | M / 44 | Hepatocellular carcinoma/IV | - | Atezolimumab; 11 days | - | Fever, hypotension, respiratory failure | mPSL, tocilizumab (8 mg/kg) | ICU admission, oxygen supplementation and intubation | 4 | 1 | N.A. | WBC 4.3 × 10^9^/L, PLT 119 × 10^9^/L, CRP 10.3 mg/L, Lactate 10.9 mmol/L, IL-6 10.1 pg/mL | Recovered |
|  | 25 | M / 84 | Transitional cell carcinoma / IV | - | Pembrolizumab; 31 days | Chemotherapy and radiotherapy | Fever, hypotension | mPSL (2 mg/kg/day, for 15 days), tocilizumab (4 mg/kg/day) | High-dose vasopressors used | 5 | 15 | N.A. | WBC 10.98 × 10^9^/L, PLT 95 × 10^9^/L, CRP 158 mg/L, Lactate 2.4 mmol/L | Recovered |
|  | 26 | M / 54 | NSCLC / IV | - | Avelumab; 2 days | - | Respiratory failure, hypotension, renal impairment | mPSL (2 mg/kg/day), tocilizumab (4 mg/kg/day) | ICU admission, oxygen supplementation, intubation, high-dose vasopressors used, HD | 5 | 0 | N.A. | WBC 21.45 × 10^9^/L, PLT 496 × 10^9^/L, CRP 129 mg/L, Lactate 11.2 mmol/L | N.A. |
|  | 27 | M / 74 | Hepatocellular carcinoma / IV | - | Nivolumab; 56 days | Chemotherapy | Fever, hypotension | mPSL (2 mg/kg/day, for 6 days); tocilizumab (4 mg/kg/day) | ICU admission, high-dose vasopressors used | 5 | 39 | N.A. | WBC 5.3 × 10^9^/L, PLT 129 × 10^9^/L, CRP 65 mg/L, IL-6 3.6 pg/mL | Recovered |
|  | 28 | F / 79 | Colorectal cancer / IV | - | Pembrolizumab; 87 days | Chemotherapy and tyrosine kinase inhibitor | Fever, fatigue, dyspnea, encephalopathy | mPSL (2 mg/kg/day, for 4 days); PSL (50 mg/day) | Oxygen supplementation | 5 | 78 | N.A. | WBC 42.33 × 10^9^/L, PLT 126 × 10^9^/L, CRP 190 mg/L, IL-6 16.9 pg/mL | Death |
|  | 29 | M / 47 | Nasopharyngeal carcinoma / IV | - | Nivolumab + ipilimumab; 26 days | Chemotherapy | Fever, confusion, hypotension (BP 74/46 mmHg), liver failure | - | High-dose vasopressors used | 5 | 25 | N.A. | WBC 19.28 × 10^9^/L, PLT 47 × 10^9^/L, CRP 67 mg/L | Recovered |
|  | 30 | M / 82 | NSCLC / IV | - | Nivolumab; 31 days | Chemotherapy and radiotherapy | Fever, arthralgia, encephalopathy | Dexamethasone (40 mg/days) | Not particular | 5 | 25 | N.A. | WBC 6.23 × 10^9^/L, PLT 206 × 10^9^/L, CRP 63 mg/L, IL-6 8.3 pg/mL | N.A. |
| Dimitriou et al. 2019 [13] | 31 | M / 47 | Melanoma / IV | - | Pembrolizumab (200mg per cycle); 4 cycles. | - | Fever, chills, rash, hypotension, tachycardia, ventricular extrasystoles, renal insufficiency, liver abnormalities | mPSL (250 mg/day for 3 days, 125 mg/day for 1 days, 75 mg/day for 1 days); PSL (50 mg/day) | Not particular | 2 | 112 | 10 | CRP 201 mg/L; IL-6 66.4 pg/mL; IFN-γ 312.7 ng/L, TNF-α 6.50 pg/mL. | Recovered |
|  | 32 | F / 48 | Melanoma / IV | - | Pembrolizumab/T-VEC (9 months);  Nivolumab/BMS-986016; 2 cycles | - | [1st.] Rash, fever, chills, tachycardia, hypotension, liver abnormalities  [2nd.] Fever, tachycardia, leukopenia, rash, stomatitis  [3rd.] Fever, tachycardia, rash, stomatitis | [1st.] PSL (80 mg/d);  [2st.] mPSL (125 mg/day);  [3st.] mPSL (125 mg/day); tocilizumab | Not particular | [1st.]3/4; [2st.]3/4; [3st.]2/4 | [1st.] 14; [2st.] 24; [3st.] 34. | [1st.] 21; [2st.] 7; [3st.] 7. | [1st.] CRP 170 mg/L, IL-6 38.4 pg/mL, IFN-γ 143.2 ng/L;  [2st.] CRP 149 mg/L, IL-6>300 pg/mL, IFN-γ>1000 ng/L, TNF-α 15.6 pg/mL;  [3st.] CRP 146 mg/L, IL-6 67.5 pg/mL, IFN-γ 2236 ng/L, TNF-α 5.73 pg/mL. | Recovered |
| Yomota et al. 2021 [14] | 33 | M / 53 | NSCLC / IVb | Diabetes and hyperlipidemia | Atezolizumab; 1 cycle | Chemotherapy and radiotherapy | Fever, rash, confusion, thrombocytopenia, lymphocytopenia, hypotension and tachycardia; disseminated intravascular coagulation | mPSL pulse therapy (1g/day, for 3 days); tocilizumab (4 mg/kg); PSL (125 mg, for 7 days); PSL (60 mg/days) | Oxygen supplementation, high-dose vasopressors used | 4 | 7 | 14 | CRP 209.1 mg/L; S-ferritin 56,520 ng/mL; IL-6 301 pg/mL | Recovered |
| Amlani et al. 2020 [15] | 34 | M / 58 | Melanoma / IV | - | Nivolumab; 180 days | - | Fever, hypotension, transaminitis, atrial fibrillation, supraventricular tachycardia, purpura, blisters/bullae, mucositis, acute kidney injury, confusion; nausea, vomiting, diarrhea | mPSL pulse therapy (1g/day, for 5 days), mPSL (150 mg/day); tocilizumab (4 mg/kg) | ICU admission, oxygen supplementation, intubation | 4 | 11 | 14 | WBC 5.7 × 10^9^/L; PLT 80 × 10^9^/L; CRP 331.4 mg/L; Ferritin 15,273 μg/L; GM-CSF 24.83 pg/mL; IFN-γ 58.19 pg/mL; IL-1β 2.91 pg/mL; IL-2 3.03pg/mL; IL-4 240.09 pg/mL; IL-5 6.71 pg/mL; IL-6 25.23 pg/mL; IL-8 68.41 pg/mL; IL-10 28.7 pg/mL; IL-12p70 2.13 pg/mL; IL-13 91.26 pg/mL; MCP-1 310.48 pg/mL; TNF-α 132.64 pg/mL | Recovered |
| Ohira et al. 2020 [16] | 35 | M / 70 | Renal cell carcinoma / IV | - | nivolumab and ipilimumab; 2 cycles. | - | Rash, muscle weakness, fever, hypotension, respiratory failure, impaired consciousness, renal failure, coagulopathy, diarrhea | PSL (60 mg/day), mPSL pulse therapy (500 mg/day, for 3days); MMF 2000mg, IVIg | ICU admission, oxygen supplementation, intubation, high-dose vasopressors used, CHDF, PE | 4 | 35 | 30 | WBC 23.9 × 10^9^/L, PLT 17.8 × 10^9^/L, CRP 240.3 mg/L, IL-1β 7.41 pg/mL, IL-2 ＜15.6 pg/mL, sIL-2R 5,764 pg/mL, IL-6 467 pg/mL, IL-10 40.6 pg/mL, IL-18 2,510 pg/mL, TNF-a 47.7 pg/mL, STNFR-1 15,400 pg/mL, STNFR-2 24,500 pg/mL, INF-γ 1,020 pg/mL | Recovered |
| Zhao et al. 2018 [17] | 36 | M / 25 | Hodgkin lymphoma / N.A. | - | Nivolumab; 1cycle | Chemotherapy | Fever, hypotension, dyspnea | - | Oxygen supplementation | 2 | 6 | 12 | ↑IFN-γ, IL-10 and CRP | Recovered |
| Ciner et al. 2021 [18] | 37 | M / 82 | Metastatic colorectal cancer / N.A. | Superficial bladder cancer | nivolumab | - | Fever, acute kidney injury, confusion, respiratory failure, hypotension | HCT (200 mg/day); dexamethasone (4 mg/day, HCT (300 mg/day, for 10 days), PSL (20 mg/day). | ICU admission, oxygen supplementation, intubation | 4 | 120 | 76 | CRP 290 mg/L | Recovered |
| Murata et al. 2022 [19] | 38 | M / 70 | Lung cancer / IIB | COVID-19 infection and hypophysitis | Nivolumab + Ipilimumab; 4 cycles | Chemotherapy | Fever, diarrhea, cardiopulmonary arrest, renal insufficiency, liver abnormalities | HCT (100 mg/day) | ICU admission, oxygen supplementation, intubation, high-dose vasopressors used, cardiopulmonary resuscitation, electrical defibrillation | 5 | 150 | 4 | WBC 9.1 × 10^9^/L, PLT 130 × 10^9^/L; PCT 29.05 ng/mL, Lactate 4.97mmol/L, CRP 102.9 mg/L, IL-6 69586 pg/mL. | Death |
| Kunimasa et al. 2021 [20] | 39 | F / 64 | Lung cancer / IVB | Smoker | Nivolumab (360 mg per cycle) and ipilimumab (1 mg/kg per cycle); 1 cycle | - | Rash, hypotension, respiratory failure | Tocilizumab (8mg/kg), infliximab (5mg/kg), mPSL/PSL, MMF | ICU admission, oxygen supplementation, intubation, high-dose vasopressors used | 5 | 67 | 43 | PCT 70.29 ng/mL, IL-6 25100 pg/mL, ferritin 1440.8 ng/mL | Death |
| Sackstein et al. 2021 [21] | 40 | M / 55 | NSCLC / IV | Hyperlipidemia | Pembrolizumab; 3 cycles | Chemotherapy and radiotherapy | Fever, hypotension, acute kidney injury, encephalopathy | mPSL pulse therapy (1g/day, for 5 days); mPSL (2mg/kg/day); tocilizumab (1st.: 8mg/kg, 2nd.: 8 mg/kg) | ICU admission, oxygen supplementation, hgh-dose vasopressors used, HD | 3/4 | 61 | 27 | Ferritin >40,000 ng/mL, ESR 85 mm/h, CRP 281 mg/L, sIL-2R 23,020 U/mL, IL-6 75 pg/mL, PLT 191 × 10^9^/L | Recovered |
| Zhang et al. 2022 [22] | 41 | F / 67 | NSCLC / IV | Hypertension | Pembrolizumab (200 mg per cycle) 1 cycles. | Chemotherapy | Fever, hypotension, hypoxemia, tachycardia, rash, liver insufficiency, acute kidney injury, and disseminated intravascular coagulation | mPSL (100 mg/day, for 3 days); PSL | Oxygen supplementation |  | 0 | 9 | WBC 17.5 × 10^9^/L, PLT 82 × 10^9^/L, CRP 57 mg/L, TNF-α 42.8 pg/mL | Recovered |
| Deng et al. 2022 [23] | 42 | F / 44 | Lung cancer / IIIC | - | Nivolumab; 1 cycle | - | Fever, tachycardia, nausea, appetite loss, liver dysfunction | Dexamethasone, IVIg | Not particular | 2 | 7 | N.A. | TNF-α 30.7 pg/mL, IL-6 45 pg/ml, IL-10 25.8 pg/ml, IL-1β 5 pg/ml, CRP 119 mg/L, PCT 0.82 ng/ml | Recovered |
| Normand et al. 2022 [24] | 43 | M / 79 | NSCLC / IV | Chronic kidney disease, chronic moderate alcohol consumption, gastroesophageal reflux disease, hypertension, ischaemic  heart disease and smoking. | Pembrolizumab (200 mg per cycle); 1 cycle | Chemotherapy and radiotherapy | Fever, confusion, dyspnoea, renal failure | Dexamethasone | Not particular | 2 | 0 | N.A. | N.A. | Recovered |
| Rassy et al. 2017 [25] | 44 | M / 62 | Squamous cell lung cancer / N.A. | Smoking | Pembrolizumab (150 mg per cycle); 8 cycles | - | Fever, diffuse swelling involving limbs and abdomen | PSL (50 mg/day, for 5 days) | Not particular | 1 | 147 | N.A. | N.A. | Recovered |
| Kogure et al. 2019 [26] | 45 | M / 67 | NSCLC / III | Smoking | Pembrolizumab; 1 cycle | - | Fever, tachycardia, hypotension | mPSL (80mg/day) | Not particular | 2 | 1 | N.A. | N.A. | Recovered |
| Hu et al. 2020 [27] | 46 | M / 50 | Colon cancer / IV | - | Nivolumab, 5 cycles; sintilimab ,2 cycles | Chemotherapy | Fever, hypotension, respiratory failure | mPSL (120 mg/day) | ICU admission, oxygen supplementation, intubation, high-dose vasopressors used | 4 | 21 | 5 | CRP 38.2 mg/L, PCT 24.76 ng/mL, INF-γ 4.77 pg/mL, IL-10 23.94 pg/mL, INF-α 4.63 pg/mL, IL-4 4.29 pg/mL, IL-6 2476.89 pg/mL, IL-2 2.59 pg/mL | Recovered |
| Gao et al. 2020 [28] | 47 | M / 69 | Esophageal cancer / IV | - | Sintilimab; 7 cycles | Chemotherapy and radiotherapy | Fever, diarrhea, nervous system injury, thyroid injury, acute renal injury | mPSL (80 mg/day); tocilizumab (80 mg); MMF (2 g) | IVIg | 2 | None reported | >12 days | IL-6 91.85 pg/mL, IL-10 9.93 pg/mL, IL-17A 5.48 pg/mL | Recovered |
| Sindel et al. 2019 [29] | 48 | F / 28 | Hodgkin's lymphoma / IV | - | Nivolumab (240 mg per cycle);1 cycle | Chemotherapy | Fever, tachycardic, hypotensive, respiratory failure | mPSL (2 mg/kg/day) | Oxygen supplementation | 2 | 35 | 11 | CRP 230 mg/L, soluble IL-2R 5739 U/mL, IL-6 52.5 pg/mL | Recovered |
| Menakuru et al. 2022 [30] | 49 | F / 58 | Melanoma / IV | - | Nivolumab and ipilimumab; 1cycle | - | Fever, tachycardic, hypotensive, respiratory failure | mPSL; tocilizumab; etanercept | ICU admission, oxygen supplementation, intubation | 4 | 6 | N.A. | CRP 20.2 mg/L, WBC 13.7 × 10^9^/L, IL-6 21.4 pg/dL, TNF-α 1,876 pg/dL | Recovered |
| Our case 1 | 50 | M / 74 | NSLC / IIIA | Liver cancer, tuberculosis, hypertension and coronary heart disease | Sintilimab (200 mg); 1 cycle | Chemotherapy | Hypotensive, respiratory failure, transaminase elevation, acute kidney injury, myocarditis, encephalitis. | mPSL (500 mg/day, for 3 days), mPSL (80 mg/day, increased to 160mg/day) | ICU admission, oxygen supplementation, intubation, high-dose vasopressors used, HD | 5 | 1 | 9 | IL-1β 11.62 pg/mL, IL-2 6.05 pg/mL, IL-2p70 12.75 pg/mL, IL-4 9.01 pg/mL, IL-5 4.18 pg/mL, IL-6 17.40pg/mL, IL-8 226.30 pg/mL, IL-10 8.57pg/mL, IL-17 40.56 pg/mL, TNF-a 10.35 pg/mL, INF-γ 9.33 pg/mL | Death |
| Our case 2 | 51 | M / 62 | Oral squamous cell carcinoma / IVA | Secondary pulmonary tuberculosis, hepatitis B virus (HBV) infection, chronic bronchitis, pneumoconiosis, hypertension and smoking | Penpulimab (200 mg); 1 cycle | Chemotherapy | Hypotensive, respiratory failure, transaminase elevation, acute kidney injury, myocarditis, atrial arrhythmia, coagulopathy. | mPSL (500 mg/day, for 3 days) | ICU admission, oxygen supplementation, intubation, high-dose vasopressors used, HD | 5 | 4 | 2 | IL-6 >5000 pg/mL, IL-10 768.83 pg/mL, PCT 6.47 ng/mL | Death |

^a^ The CRS grade was based on the Lee et al. Blood. 2016;128(11):1533. grading scale.

WBC, white blood cells; PLT, platelets; CRP, C-reactive protein; PCT, procalcitonin; T-VEC: talimogene laherparepvec; ICU, intensive care unit; IVIg, intravenous immunoglobulin; MMF, mycophenolate mofetil, mPSL, methylprednisolone; PSL, prednisolone; HDC, hydrocortisone; CHDF, continuous hemodiafiltration; HD, hemodialysis; PE, plasma exchange; IL, interleukin; TNF-α, tumor necrosis factor-alpha; IFN-γ, interferon gamma; STNFR, soluble tumor necrosis factor receptor; N.A., Not applicable.
